# Emergency Medicine Scholarly Tracks: A Mixed- methods Study of Faculty and Resident Experiences

**DOI:** 10.5811/westjem.19453

**Published:** 2025-07-10

**Authors:** Jason Rotoli, Ryan Bodkin, Grace VanGorder, Valerie Lou, Lindsey Picard, Beau Abar

**Affiliations:** * University of Rochester, Department of Emergency Medicine, Rochester, New York; † Penn State College of Medicine, Hershey, Pennsylvania

## Abstract

**Objectives:**

Emergency medicine (EM) scholarly tracks have been adopted for increased subspecialty exposure and training. However, current literature fails to elucidate the impact on faculty and resident careers and resident and faculty engagement opportunities or demonstrate barriers to continuation. The purpose of this study was to evaluate the perceived impact of EM scholarly tracks on participating faculty (eg, resident interaction/mentorship, career satisfaction, perceived barriers to implementation) and recent graduates (eg, faculty mentorship, reasons for track selection, perceived barriers to continuation).

**Methods:**

This mixed-methods study includes a cross-sectional quantitative survey with 30 EM residents (who graduated between 2021–2023) and semi-structured, one-hour qualitative interviews with six faculty in a large, tertiary-care academic medical center with a university-based hospital and medical school. We conducted frequency analyses on demographics, timing of tracks, mentorship impact, and implementation barriers. Chi-square analyses were used to compare the most and least common reasons for track selection. We evaluated faculty data in a program evaluation framework, seeking commonalities and idiosyncratic experiences.

**Results:**

**Resident Data:**

Most participants pursued either academic or hybrid academic/community careers (18/30). Additionally, most participants reported a positive impact on mentorship (25/30). The most common reason for choosing a track was “area of clinical interest” (mean 2.93, *P* <.001). The least common reason was “lowest effort/amount of work” (mean 1.47, *P*<.05) when compared to half of the other choices. Most residents did not report barriers to track continuation.

**Faculty Data:**

Faculty frequently discussed how resident scholarly tracks led to increased one-on-one faculty: resident time. Additionally, they reported the opportunity for specialization of residents not seeking fellowships. A reported barrier to continuation of and resident engagement in tracks was the balance needed between teaching enough and over-teaching, which can discourage learner interest.

**Conclusion:**

Recent EM graduates and current faculty members participating in scholarly tracks reported a positive impact on engagement and mentorship with minimal reported barriers to implementation and continuation. Scholarly tracks may offer more than educational benefits to participants, including individualized mentorship and career guidance.

## INTRODUCTION

Since emergency medicine (EM) was approved as a primary medical specialty by the American Board of Medical Specialties in 1989, opportunities for subspecialty training have gradually increased. Currently, there are seven fellowships recognized by the Accreditation Council for Graduate Medical Education (ACGME), 11 fellowships recognized by the American Board of Emergency Medicine (ABEM) (Table 1), and many more non-ACGME/ABEM fellowship training opportunities available to emergency physicians under other board specialties.^1^–^3^ In 2017, a survey of US EM program directors found that approximately 40% of EM residency programs had subspecialty tracks available to trainees.^4^ Track format varied widely, including required didactics, experiential learning models, and longitudinal electives.

**Population Health Research Capsule**

What do we already know about this issue?

*Data show the benefits of subspecialty tracks, but no study has looked at the perceived benefits for both residents and faculty on a more granular level*.

What was the research question?

*We evaluated the perceived impact of tracks on participating faculty and recent EM graduates*.

What was the major finding of the study?

*Subjects mostly pursued either an academic or hybrid academic/commmunity care career (18/30) and most reported a positive impact on scholarly tract mentorhip (25/30)*.

How does this improve population health?

*Scholarly tracks can lead to enhanced career satisfaction for both EM faculty and residency graduates, which in turn could result in better care for their patients*.

A few studies have assessed the efficacy of EM track-based education and the association with residents’ interest in fellowships.^5^–^8^ Scholarly track-based learning is associated with pursuing a career in academic EM, and residencies often integrate tracks to enhance career guidance by inspiring residents to choose academic careers.^7^ However, most of the studies that have looked at the benefits of tracks, such as faculty development and mentorship, are anecdotal and based on consensus literature from program director surveys. Research that directly assesses the perceived impact on participating EM residents and faculty is sparse.^7^,^9^ Additionally, while some research describes best practices when implementing EM tracks and suggests why tracks may not exist (ie, lack of time, lack of faculty, limited administrative support), little research has investigated barriers to the continuation of EM subspecialty tracks after they have been established.^4^,^9^ We aimed to evaluate how participating faculty members and recently graduated residents perceived track-based education. Given the benefits of robust mentorship for EM residents and faculty (eg, career success, professional growth, academic productivity, and opportunity to give back to the profession), we hypothesized that academic faculty and residents would report improved engagement (mentorship) opportunities and enhanced career satisfaction.^10^,^11^ Additionally, we investigated EM residents’ perceived barriers to track continuation.

## METHODS

### Study Design and Survey

We conducted this mixed-methods study, comprised of a cross-sectional quantitative survey of 30 EM graduates and semi-structured qualitative interviews with six faculty at an academic medical center in the northeastern US with a university-based hospital, a medical school, and >100,000 annual ED patient visits. We defined a career in academics as working in a medical center with a university-based hospital, university ownership/affiliation, and/or an affiliated medical school. Alternatively, we defined a career in community EM as working in a hospital with limited (or no) relationship with a university/medical school and with a limited numbers of (or no) learners. A hybrid career was defined as working any amount of time in both academic and community settings. Our department has a three-year, ACGME-accredited program with 14 residents per class, seven fellowships with 8–10 fellows per year, and approximately 80 faculty members.

We developed quantitative and qualitative survey questions using the process of iteration and literature review for research gaps, and by harnessing the research team’s academic experience and experience with national grant funding, EM fellowship training (medical education), and residency administration. The team consisted of one medical student interviewer, two junior academic faculty, two senior academic faculty, and one research methodologist. Collectively, and through an iterative process, we capitalized on team expertise to design the survey, which we refined and subsequently pre-tested for errors and comprehension. While we did not formally assess construct validity, the survey was reviewed by an external content expert and the study team for face validity.^12^ Although less widely accepted than construct validity, face validity is a complex paradigm used to evaluate how respondents perceive test items. It consists of many dimensions, including accuracy, acceptance (likeability) and relevance, and has been regarded as an acceptable form of validity in prior medical education curricula.^13^–^15^

We collected and managed survey data using Research Electronic Data Capture (REDCap) tools hosted at the University of Rochester. The survey questions sent via email to EM graduates included demographics (age, graduation year, completion of fellowship, career selection), critical review of track implementation (reasons for track selection, timing, barriers), and training impact (mentorship opportunities). They answered questions using a three-point Likert scale with a “strongly negative,” “neutral,” or “strongly positive” response (see Appendix A). The faculty data came from one-hour, semi-structured interviews conducted by a third-party person trained by a research team member. The faculty interviews targeted four areas: 1) demographics (additional degrees, years at the institution, scholarly track role such as director, creator); 2) resident interaction (opportunities for mentorship, engagement, and non-clinical evaluation); 3) impact on career (longevity, trajectory, opportunities for scholarly work); and 4) barriers to track perpetuation (See Appendix B).

Faculty were interviewed using a program evaluation framework, seeking evidence of common and distinctive feedback to improve the experience of the tracks program. We designed the interview questions to address tier 3 (understanding and refining the program), tier 4 (continuing progress toward desired outcomes), and tier 5 (broad program impact) of the five-tiered approach to program evaluation (Figure).^16^ To maintain anonymity in feedback, interviews were not recorded or transcribed. Instead, the interviewer took notes on faculty responses, requesting confirmation from participants when summarizing or quoting. To further maintain confidentiality we did not collect faculty-associated tracks.

### Recruitment, Consent, and Risk to Subjects

#### Resident Selection and Recruitment

Since our track learning began in 2019, inclusion for the study included any residents who graduated between 2021–2023. Exclusion criterion was any resident who did not graduate within this period. Eligible participants were emailed information detailing the project’s objectives. Participation was voluntary. The email contained a link to the REDCap survey. All data collection was anonymous. Completion of the survey implied consent. We sent a reminder email two weeks after the initial email to maximize participation.

#### Faculty Selection and Recruitment

Inclusion for the study included any faculty deemed a scholarly track leader or creator. We excluded faculty who did not lead or create a scholarly track. Our goal was to obtain a representative sample with respect to tracks supervised, stage of academic career, and faculty sex. We emailed eligible faculty an information sheet that detailed the project’s objectives and emphasized that participation was voluntary. We sent reminder emails two weeks and four weeks after the initial email. Interview scheduling and completion implied consent.

There were no sex, race, or ethnicity-based restrictions. This project was undertaken as a quality improvement initiative. Per the University of Rochester’s Guideline for Determining Human Subject Research, it did not meet the definition of research according to 45CFR46 and was exempt from institutional review board-approval. Data were anonymous for all participants, and there was minimal risk to participant confidentiality.

#### Track Structure

The tracks start at the beginning of postgraduate year (PGY)-2 year and culminate at the end of the PGY-3 year. The sessions, 60–90 minutes long, take place during didactic conference 9–10 times per academic year. Tracks are led by 1–3 faculty members and have a maximum of 1:3 faculty-to-resident ratio. Track curricula are 18–20 months long and incorporate required elements that are the same across all tracks, including basic research certification training, a medical student- or peer-teaching requirement, and choosing a research question. Other required elements are unique to each specific track, including didactics, journal clubs, community engagement (eg, partnering with local shelters to organize an activity), track-specific certifications (eg, advanced research training), or hands-on activities (eg, joining a Wilderness Medicine Society or local- event medicine). Similarly, the requirements for track completion vary based on the chosen subspecialty but with some overlap for all tracks (eg, teaching component and research projects). Given the intensity of the PGY-2 year in a three-year training program, residency leadership backloaded many of the requirement due dates to the end of PGY-2 and early/mid PGY-3 year. For residents who have any interests outside existing tracks, the “Education Track” empowers them to pursue these interests.

### Analysis

We conducted frequency analysis on resident demographics, mentorship impact, timing of tracks, most common track choice, and implementation barriers. Chi-square analyses were used to compare reasons for resident track selection. The initial evaluation of faculty interviews was conducted by a non-clinical, research faculty team member (BA) in a program evaluation framework, seeking commonalities and distinctive experiences that might inform track revision. It has been reported that using deductive and inductive analytic practices provides the deductive tools to organize the data, allows findings to emerge from the data, and applies existing knowledge and theory to interpret and explain findings.^17^ We used inductive coding and constant comparative analysis, which enabled us to analyze the responses to discover common themes among the data.^18^,^19^ Due to limited numbers of participants, data were insufficient for more robust analysis methods, such as grounded theory analysis.

## RESULTS

### Resident Data

The response rate for the resident survey was 71% (30/42). Most participants were male (19/30) (corresponding to the demographics of the sample pool of residents), between 30–34 years of age (23/30), and had either academic or hybrid (academic/community) careers (18/30). A minority of participants had purely academic EM jobs (6/30) and one third (10/30) of all graduates had either completed or were completing a fellowship, with ultrasound the most common (4/10).

Eighty-three percent (25/30) of participants reported a positive impact on mentorship during residency. The most common reason for track selection was “area of clinical interest” (93% report this reason as “very important” (overall chi-square and each subsequent pairwise comparison <.001)). Most residents did not report barriers to track implementation and continuation, with only one third of residents (10/29) reporting that COVID-19 interfered with scholarly track implementation. (Table 2 lists complete results.)

### Faculty Data

The response rate for the faculty interview was 75% (6/8). Most faculty members frequently discussed how the implementation of tracks led to increased one-to-one faculty-resident time and engagement with residents and provided opportunities to guide and observe resident skill development outside the clinical arena. One faculty member commented how faculty can use “[track] relationships to form new, deeper connections— to see a different way of [resident] thinking.” Three faculty members discussed the opportunity that tracks provide for education in hands-on skills that are difficult to teach in large group settings, particularly to learners who are not particularly interested in the area of instruction (Table 3). Additionally, faculty favorably reported that tracks offered the opportunity for some specialization for non-fellowship seeking residents. For example, one faculty member noted, “[tracks] allow teachers to talk more about a specific advanced topic of interest and provide enthusiastic residents with a focused learning curriculum outside of the group setting.” Shared and distinctive themes and supporting comments can be found in Table 3.

When discussing barriers to continuation, faculty suggested striking a balance between teaching too much to discourage interest and teaching enough to engage interested learners during a track meeting. There was no thematic saturation regarding the impact on faculty career longevity.

## DISCUSSION

Among many factors in the complex decision of choosing a career, scholarly tracks have been associated with a higher likelihood of an academic career choice.^8^,^20^ Although not directly investigated in this study, most graduates in our study worked in academic or hybrid careers.^5^ Additionally, one-third of the participants entered or completed academic fellowships, consistent with Jordan et al’s reported percentages of track-trained residents entering fellowships in 2018.^5^ Both results are logical, as scholarly track training can inspire residents to choose fellowships, and fellowship-trained graduates may be more likely to look for jobs and are often sought out by academic institutions. As supported by a study by Jordan et al in 2024, track exposure is one of several factors that guide residents down their career paths as it increases exposure to and involvement with faculty scholarly work and mentorship.^20^

On the contrary, an older study from 2008 contradicts our findings, reporting only that 23% of residents accept academic jobs and 5% fellowship entry. This discrepancy may be due to the Lubavin study design; they included community- and academically-trained EM residents, with community-trained EM residents less likely to pursue academic EM careers. Additionally, the 16-year time difference between that study and ours reflects potentially more contemporary motivators to pursue academics, including more fellowship options and an increased emphasis on wellness through balance of clinical and academic (research, leadership, teaching, and mentorship) roles.^8^,^21^,^22^

Over 80% of our graduated residents noted the increased opportunities for mentorship. Given the excellent faculty-to-resident ratio, additional time for interaction, and shared passion for an academic interest, this is one of the most important aspects of track training. Although nearly all the previous EM program director literature has perceived this and it has been documented by participating residents in other specialties, to our knowledge this is the first study with direct feedback from EM residents who participated in tracks during residency.^23^,^24^

The most common reason for track choice was “area of clinical interest.” This is a logical finding, given the increased desire to learn from like-minded people with common interests and goals. The least common reason for track selection was “lowest effort/amount of work.” In an academic residency, this finding may be related to residents being more motivated to pursue scholarly work during their training. To our knowledge, these motivations have not been measured in participating EM residents until now.

Overall, most residents did not report barriers to track continuation. Given the structure of the tracks (eg, backloaded due dates to avoid adding to high-intensity training periods and dedicated time during didactics), there was minimal reported impact on clinical obligations or work-life balance. Additionally, resident participation and interest were not reported as barriers to track continuation. This may have been influenced by the program’s dedication and faculty interest in track implementation. Also, subspecialty tracks are not new, and we likely benefited from previously published literature on the perceived benefits, as well as pitfalls to avoid when creating tracks. To our knowledge, these have not been measured directly by participating EM residents until now.

Participating faculty had a favorable view of the impact of the subspecialty track on interaction with residents. The faculty reported increased time with residents and the potential to inspire and educate non-fellowship seeking residents. Although this study did not establish causality, this additional time and mentorship from participation in subspecialty tracks are consistent with current literature as two of the many factors that help guide graduates in their career-decision process and to stay focused on academic careers.^20^ Also, faculty used track sessions to observe non-clinical skill development, including time management, project execution, and communication. This allowed for a deeper understanding of the residents’ development, which fostered enhanced career guidance through more individualized coaching and mentorship. One reported barrier to the continuation of tracks was the need to establish a balance between teaching and “over-teaching” to keep learners engaged and cultivate curiosity but prevent them from feeling overwhelmed. Notably, overwhelming learners has been shown to reduce active learning/engagement and has proven to be time-consuming (and detrimental) for the instructor.^25^

## LIMITATIONS

First, this study measured recently graduated EM resident perceptions of track impact over a limited period (three years) and did not measure impact directly. Additionally, we did not have access to comparative data from the three years preceding the establishment of tracks, making it insufficient to establish causation. Second, the study was conducted at a single academic institution, potentially limiting generalizability to smaller (or community) institutions without a robust core faculty with dedicated time for or expertise and interest in track implementation. Additionally, the lack of standardization of implementation and design of educational tracks across programs makes it difficult to generalize our results to other institutions. Also, single-center studies can overestimate effect in comparison to multicenter studies.^26^ Third, a limited set of faculty members provided qualitative feedback about the program, and a single research team member was responsible for collating responses in a program evaluation framework. Subsequent evaluation work might benefit from a larger faculty sample and the use of more extensive qualitative analysis methods (eg, transcription, grounded theory analysis).^27^

Fourth, pre-selection bias may have influenced our results as residency applicants who are interested in pursuing subspecialty fellowships may have initially chosen our residency program due to existing fellowships. Consequently, these trainees may have been more likely to positively review and participate in scholarly tracks. Fifth, self-selection bias may have influenced qualitative results, given that the faculty chosen to participate may have been more apt to favor the scholarly track work and highlight its benefits. It may have also influenced some of the resident data; specifically, the least common reason for choosing a track was “lowest effort/amount of work,” as recently graduated residents may not have wanted to be perceived as lazy or uninterested in learning during a study conducted by former colleagues/mentors. Lastly, non-response bias may have been present; it is important to acknowledge that any patterns uncovered in analyzing a non-random sample do not provide valid grounds for generalizing about a population.^28^,^29^

## CONCLUSION

Recently graduated EM residents reported that scholarly tracks positively impacted mentorship. While this study did not assess causation between tracks and academic careers, most participating residents chose academic careers and reported few implementation barriers. The impact on faculty engagement (mentorship) with residents was reported to be high. Participating faculty reported a more holistic view of resident development, suggesting that scholarly tracks may offer more than educational benefits to participating residents, including individualized mentorship and career guidance. This work was conducted at a single site, and track-related education is dynamic, both locally and at other EM residencies. Future multisite research should build upon our evaluative work by investigating potential causation of track integration on resident career choice including comparative data analyzing pre- and post- track implementation.

## Supplementary Information







## Figures and Tables

**Figure:**
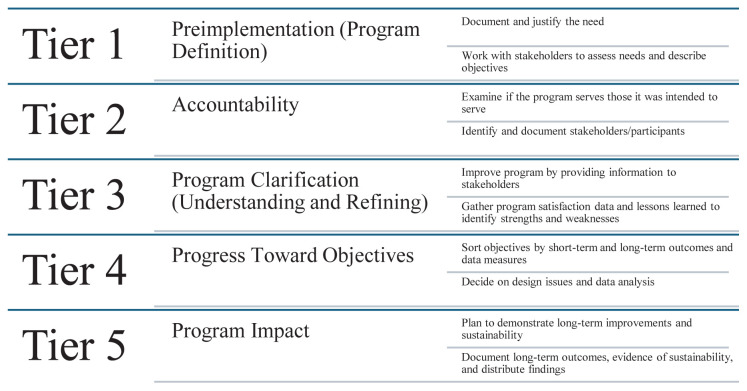
Five-tiered approach to program evaluation.^10^

**Table 1:** Subspecialty training tracks available to emergency medicine residents.

American Board of Emergency Medicine EM Subspecialties	Accreditation Council for Graduate Medical Education (ACGME) EM Subspecialties	Non-ACGME EM Subspecialties
Anesthesiology Critical Care Medicine Emergency Medical Services Healthcare Administration, Leadership and Management Hospice and Palliative Medicine Internal Medicine Critical Care Medicine Medical Toxicology Neurocritical Care Pain Medicine Pediatric Emergency Medicine Sports Medicine Undersea and Hyperbaric Medicine	Addiction Medicine Clinical Informatics Emergency Medical Services Medical Toxicology Pediatric Emergency Medicine Sports Medicine Undersea and Hyperbaric Medicine	Addiction Medicine Aerospace Medicine Emergency Ultrasound Wilderness Medicine

*EM*, emergency medicine.

**Table 2:** Resident perceptions of the scholarly education tracks in emergency medicine.

	Strongly Disagree	Disagree	Agree	Strongly Agree
The scholarly EM tracks positively impacted mentorship from EM faculty	0 (0%)	5 (17%)	15 (50%)	10 (33%)

	0–30Minutes	31–60 Minutes	61–90 Minutes	91–120 Minutes

How much didactic time per month should be dedicated to EM scholarly tracks?	4 (13%)	11 (37%)	11 (37%)	4 (13%)

EM Track	Initial Choice^a^	Would choose a different track if could do it again^a^	Would have switched to this track^a^	

Research	2 (7%)	1 (3%)	0	
Toxicology	3 (10%)	1 (3%)	1 (3%)	
Education	9 (30%)	3 (10%)	0	
EMS	4 (13%)	2 (7%)	0	
Ultrasound	5 (17%)	1 (3%)	3 (10%)	
Pediatric Emergency Medicine	4 (13%)	2 (7%)	0	
Wilderness Medicine	3 (10%)	0 (0%)	1 (3%)	
Critical Care	0 (0%)	0 (0%)	4 (13%)	
Global Health	0 (0%)	0 (0%)	1 (3%)	

Reason for Initial Track Choice	Not Important	Somewhat Important	Very Important	

Self-identified area of clinical weakness	12 (40%)	14 (47%)	4 (13%)	
Area of clinical interest	0 (0%)	2 (7%)	28 (93%)	
Fellowship preparedness	15 (50%)	9 (30%)	6 (20%)	
Job market competitiveness	15 (50%)	9 (30%)	6 (20%)	
Aligned with previous academic interest (before residency)	7 (23%)	12 (40%)	11 (37%)	
Obligation to choose a track	11 (37%)	14 (47%)	5 (17%)	
Lowest effort/amount of required work^c^	18 (60%)	10 (33%)	2 (7%)	

Perceived Barriers to EM Tracks	Yes	No		

Insufficient faculty support	4 (13%)	26 (87%)		
Insufficient scholarly track options	7 (23%)	23 (77%)		
Insufficient resident participation	4 (13%)	26 (87%)		
Insufficient resident interest	5 (17%)	25 (83%)		
Conflicted with other academic obligations	8 (27%)	22 (73%)		
Conflicted with clinical obligations	6 (20%)	24 (80%)		
Conflicted with work-life balance	5 (17%)	25 (83%)		
COVID-19 pandemic^b^	10 (33%)	20 (67%)		

a Initial choices and interest in switching tracks was partially influenced by new tracks offered to later cohorts.

b Potential for differential perceptions of COVID-19 pandemic was influenced by residency cohort timing.

c Paired-samples comparisons showed that scores on the lowest effort/amount of required work item were significantly lower than the (a) area of clinical interest, (b) aligned with previous academic interest, and (c) obligation to choose a track score, all *P*-values < .05.

*EM*, emergency medicine; *EMS*, emergency medical services.

**Table 3:** Selected faculty qualitative feedback regarding scholarly tracks programs in emergency medicine.

**How do you feel involvement with tracks has affected your interaction with residents?**	
More 1:1 time with residents in their track (4/6)	“…Helped residents identify areas of interest and give them the opportunity to explore those.”
Opportunity for greater depth of training (3/6)	“…Allow teachers to talk more about a specific advanced topic of interest.”
Benefits those who are motivated (4/6)	“Provide enthusiastic residents with a focused learning curriculum outside of the group setting”
	“If a resident is motivated, track can provide an additional benefit of guidance. This is a specific [type of resident], and not all residents will benefit from the tracks if they are not motivated to learn more in a specific area”
Deeper relationships with residents (3/6)	“[allow us to] form new, deeper connections.”
	“Get to know residents outside of medical setting.”
Allows for better evaluation of residents (1/6)	“…see skills outside of clinical progress… [can] use track relationships to help with choice of chief resident.”

**How has involvement with tracks changed your opportunity for mentorship?**	
Focus on fellowship-interested students (1/6)	“We can [guide] these students to be prepared for this fellowship... They can see the career trajectory in reality and be guided in next steps. [They get a] realistic understanding of what a career in [redacted] looks like.”
Opportunity for focused, hands-on mentorship (3/6)	“Harder to teach [redacted] to large group because it is a hands-on skill.”
More focused career guidance (3/6	“[Offers] ability to talk about my career [path].”
Greater resident collaboration and networking (4/6)	“Increased opportunities for residents to network with each other (connection between second and third years, which doesn’t always happen without tracks) … “Residents become closer to each other. [They] have their own group chats.”

**How has involvement with tracks affected how you feel about your career in academic EM?**	
No impact (3/6)	“It has had no effect. More beneficial to junior faculty and fellows to find [their] teaching style or work on research projects.”
Increased satisfaction (3/6)	“Being involved in something else has helped prevent burnout.”
	“Adds variety [and] shifts focus outside of clinical work.”
	“[Incorporation of] tracks has made work fun.”

**How has involvement with tracks affected your career trajectory or anticipated career longevity?**	
None (4/6)	
Expanded scope (1/6)	“Added mentorship and teaching into clinical medicine.”
Extended duration (2/6)	“Has added years to career due to burnout prevention.”
	“I stay up to date because [I am learning] alongside the residents about changes in the field.”

**How has involvement with tracks changed the level of involvement in other professional activities?**	
Tradeoffs due to time commitments (2/6)	“Juggling schedule between med students, residents, clinical schedule and teaching [is challenging].”
	“Time consuming meeting with residents, preparing learning materials and objectives”…

**Were there barriers to implementation and participation that diminished your overall experience?**	
Too many residents in their track (1/6)	“Originally believed as a track leader, I would be responsible for guiding residents through QA and research projects. I cannot possibly do this for [a high number of’] residents.”
Scheduling difficulties in tracks (1/6)	“Sometimes, it is difficult to schedule events as having 1–2 residents on vacation makes the group too small to invite a guest lecture or to plan an event.”
None (3/6)	

**Additional Feedback Offered**	
Long-term planning of activities needed (2/6)	“Want to see a curriculum that is 24 months in the making—residents and faculty can know what is coming far in advance.”
	“Must have structure. By having a structured program, the track program will provide benefit to the residents instead of being another box they must check.”
Tracks should not be mini-fellowships (2/6)	“Want to find balance between teaching those who want to learn and not discouraging learners from pursuing fellowship by teaching too much.”

Note: Fractions in left column indicate how many faculty members expressed this theme. [Redacted] indicates EM track or division that was eliminated from the text for sake of participant anonymity.

*EM*, emergency medicine.
