# Supplementary material for: Emergency Medicine Scholarly Tracks: A Mixed- methods Study of Faculty and Resident Experiences
Source: West J Emerg Med. 2025 Jul 10;26(4):786–94. doi: 10.5811/westjem.19453 (PMC12342406; doi:10.5811/westjem.19453)
Supplement: Supplementary file 1 [file wjem-26-786-g001.pdf]

Tier 1	Preimplementation (Program Definition)	Document and justify the need<br>Work with stakeholders to assess needs and describe objectives
Tier 2	Accountability	Examine if the program serves those it was intended to serve<br>Identify and document stakeholders/participants
Tier 3	Program Clarification (Understanding and Refining)	Improve program by providing information to stakeholders<br>Gather program satisfaction data and lessons learned to identify strengths and weaknesses
Tier 4	Progress Toward Objectives	Sort objectives by short-term and long-term outcomes and data measures<br>Decide on design issues and data analysis
Tier 5	Program Impact	Plan to demonstrate long-term improvements and sustainability<br>Document long-term outcomes, evidence of sustainability, and distribute findings
