# Supplementary material for: Emergency Medicine Scholarly Tracks: A Mixed- methods Study of Faculty and Resident Experiences
Source: West J Emerg Med. 2025 Jul 10;26(4):786–94. doi: 10.5811/westjem.19453 (PMC12342406; doi:10.5811/westjem.19453)
Supplement: Supplementary file 2 [file wjem-26-786-s001.docx]

*Appendix - Track Alumni Survey*

**Survey on Tracks**

Please complete the survey below. Thank you!

What is your age (in years)? 25-29

30-34


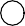

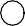

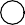

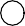

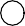


35-39

40-44

>44

What is your gender? Female

Male


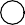

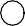

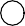

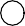


Other/Non-Binary Prefer not to answer

What year did you graduate EM residency? 2020

2021


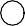

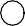

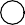

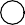


2022

2023

Do you work in...? Community EM

Academic EM


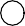

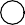

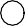


Combination of Community and Academic EM

What is your current academic title? Clinical Instructor

Senior Clinical Instructor Assistant Professor Associate Professor Professor


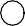

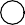

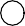

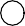

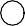


The scholarly EM tracks positively impacted mentorship Strongly Disagree from EM faculty Disagree

Agree


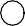

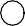

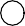

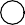


Strongly Agree

When reflecting on the amount of time that was Too little

dedicated to EM scholarly tracks, do you think that Just right


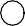

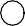

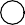


the time was... Too much

How much didactic time per month should be dedicated 0-30 minutes to EM scholarly tracks? 31-60 minutes

61-90 minutes


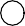

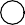

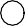

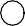


91-120 minutes

What track did you choose? Research

Toxicology Addiction Medicine Education


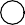

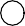

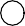

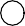

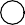

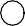

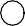

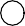


EMS

Ultrasound

Pediatric Emergency Medicine Wilderness Medicine

If you could do it again, what you choose a different No

subspecialty track? Yes


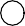

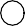


Which track would you choose? Research Toxicology Addiction Medicine Education


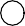

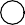

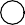

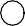

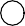

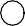

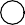

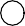

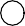

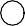

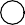

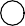

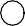

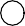

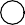


EMS

Ultrasound

Pediatric Emergency Medicine Wilderness Medicine

Biopsychosocial Emergency Medicine Critical Care

Hyperbarics/Wound Care Aerospace Medicine Global Health

Healthcare Administration Other

Describe other

Did you complete or are you currently completing an EM Yes fellowship? No

Which one? Research

Toxicology Addiction Medicine Education


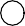

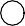

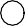

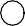

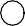

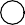

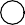

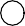

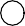

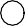

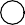

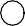

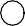

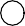

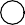

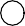

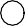


EMS

Ultrasound

Pediatric Emergency Medicine Wilderness Medicine

Biopsychosocial Emergency Medicine Critical Care

Hyperbarics/Wound Care Aerospace Medicine Global Health

Healthcare Administration Other

Describe other

**How important were the following when you chose your track?**

Not important Somewhat important Very important

Self-identified area of clinical
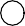

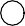

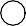
 weakness

Area of clinical interest
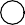

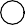

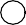
 Fellowship preparedness
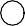

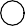

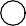
 Job market competitiveness
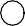

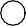

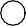


Aligned with previous academic
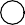

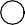

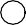
 interest (before residency)

Obligation to choose a track
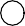

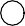

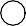


lowest effort/amount of required
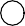

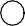

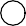
 work

**Were any of the following barriers to successful implementation? yes/no (may choose more**

**than one)**

No Yes


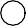

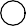

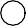

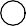
Insufficient faculty support
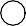
 Insufficient scholarly track options Insufficient resident participation

Insufficient resident interest

Conflicted with other academic obligations

Conflicted with clinical obligations

oConflicted withwork-life balance COVID-19 pandemic

other

Any additional feedback/comments you would like to share regarding your experience with EM tracks
