# Supplementary material for: Emergency Medicine Scholarly Tracks: A Mixed- methods Study of Faculty and Resident Experiences
Source: West J Emerg Med. 2025 Jul 10;26(4):786–94. doi: 10.5811/westjem.19453 (PMC12342406; doi:10.5811/westjem.19453)
Supplement: Supplementary file 3 [file wjem-26-786-s002.docx]

**Appendix - Qualitative Interview Guide**

**Demographic Information**

1. How many years have you been practicing Emergency Medicine (EM) as an attending?
2. How many years have you been EM faculty at the University of Rochester Medical Center (URMC)
3. Which sites under the URMC umbrella do you currently practice?
4. What is your academic title?
5. Are you core faculty?
6. Are you fellowship trained? If so, what fellowship?
7. Do you have any additional degrees or training? If so, what are they?
8. Besides clinical care and teaching, what other role(s) do you fulfill at URMC?
9. Which track(s) are you involved with? What is your role (director, co-director, contributing faculty)

**Resident Interaction**

- “Probe” = only if needed (do not start with these inquiries)

1. How do you feel involvement with tracks has affected your interaction with residents?
   1. Probe (better/worse)*:
      1. Can you explain why/how?
   2. Probe (if they are not talking)*:
      1. Does it change your relationships with the residents?
      2. Are the residents more engaged with you?
2. How has involvement with tracks changed your opportunity for mentorship?
   1. Probe*:
      1. Increased or decreased opportunities
      2. More career guidance
      3. More group vs individual mentorship
      4. Increased opportunity to help residents network
3. Do you have resident mentees?
   1. If yes: Approximately what proportion of your current mentees share the same track interest that you do?
   2. How has this proportion changed over time?

**Faculty Career Satisfaction**

1. How has involvement with tracks affected how you feel about your career in academic EM?
2. How has involvement with tracks affected your career trajectory?
3. How has involvement with tracks affected your anticipated career longevity?
4. How has involvement with tracks changed the level of involvement in other professional activities?

**Barriers to implementation**

1. Were there barriers to implementation and participation that diminished your overall experience?
   1. Prompt (time, salary support, too many or too few participants)
2. Did COVID-19 create a barrier to implementation?
   1. If yes, how?
